# Interaction of Some Cultural Practices and Reduced Herbicide Rates Used for Water Conservation Through Weed Control

**DOI:** 10.3390/plants15213336

**Published:** 2026-10-28

**Authors:** Zvonko Pacanoski, Gordana Glatkova, Biljana Drvoshanova, Katerina Bandjo Oreshkovikj

**Affiliations:** 1 Faculty of Agricultural Sciences and Food—Skopje, Ss. Cyril and Methodius University in Skopje, 16ta Makedonska Brigada 3, 1000 Skopje, North Macedonia; 2 Institute of Agriculture, Ss. Cyril and Methodius University in Skopje, 16ta Makedonska Brigada 3A, 1000 Skopje, North Macedonia; g.glatkova@zeminst.ukim.edu.mk (G.G.); b.drvoshanova@zeminst.ukim.edu.mk (B.D.); k.bandzo@zeminst.ukim.edu.mk (K.B.O.)

**Keywords:** weeds, cultural practices, water conservation, weed control, reduced herbicide rates

## Abstract

In the context of increasing agricultural plant productivity and crop yields, water losses due to weeds pose a significant constraint. Therefore, this review aims to examine weed-induced water losses and evaluate the potential of various cultural practices (mulching, green manures, cover crops, crop rotation, and intercropping) in combination with reduced herbicide rates for sustainable soil water conservation through effective weed management. The published literature indicates that surface mulches (plastic and organic layers) and cover crop residues exhibit the strongest empirical support for simultaneous weed suppression and direct evaporation reduction. The duration of weed control depends heavily on biomass composition, where high-lignin biomass extends the active physical suppression window compared with rapidly degrading, low-lignin tissues. Integrating these cultural practices with low-input herbicide programs makes a 30% to 50% reduction in pre- and post-emergence herbicide rates highly realistic under field conditions without compromising crop yields. Finally, this review addresses critical agronomic trade-offs, as well, such as physical herbicide interception by residues, and highlights future horizons for pairing these integrated cultural tactics with advanced precision weed-management technologies to secure long-term agroecosystem resilience and soil water conservation.

## 1. Introduction

One of the biggest challenges in agriculture is the management of water, as it is widely considered the greatest limiting resource for crops [1,2]. This limitation is especially important and evident in arid and semi-arid environments [3,4]. Crops consume large quantities of water [5], and most of it is lost by transpiration to the atmosphere. On the other hand, weeds directly compete with crops for water, limiting its availability for crop growth [6]. Limited soil water availability influences crop–weed competition to a significant degree, and, in general, weed growth is favored due to the greater plasticity of weeds as compared with crops [7,8]. Accordingly, weeds consume water intended for crops, cut water availability, and contribute to crop water stress [9,10]. Particularly, during years of water shortage, when moisture is in short supply, weeds can reduce crop yields by more than 50% through moisture competition alone [11]. In that context, growth of *Chenopodium album* L. (common lambsquarters), *Kochia scoparia* L. (common kochia), and *Salsola tragus* L. (Russian thistle) is less affected by a shortage of moisture than is the growth of many crops [12]. Generally, weeds are known as great water wasters [13,14,15]. The roots of some weeds, such as *S. tragus*, develop much faster than roots of the crop with which it is competing. This allows the faster-developing weeds to reach deeper soil moisture first [16].

Water conservation is defined as minimizing the loss or waste, caring for and protecting water resources, and the efficient use of water [17,18]. There are many ways to conserve water. One of them is effective weed control. Mulching is the practice of covering the soil surface to make favorable conditions for plant growth and development. The main objective of mulching is water saving and weed control [19,20]. Mulches, when spread over the soil surface, minimize water runoff [21], increase infiltration [22], provide shade to the soil (suppress weeds) [23], and act as a barrier to reduce water loss in the form of vapors [19]. Green manures cover the soil surface [24], blocking light and altering the temperature and moisture at the soil surface [25] in ways that can impede weed germination or interfere with weed growth [26]. Some green manures are also allelopathic, containing or secreting compounds that can inhibit the germination of the weeds [27,28]. Weed control through cover crop management has several impacts on soil properties and affects soil and water conservation due to changes in soil structure in the row and interrow crop [29,30]. Cover crops and cropping residues serve as protection for the soil surface against weather aggression and water erosion [31], to maintain soil moisture [32], suppress weed growth [33], and provide shelter and food for the soil biota [34]. According to Meschede [35], besides the allelopathic effects, proper use of cover crops can provide control of weed plants by altering several system features, such as thermal regimes, incidence of light and physical barriers to emergence, and also increasing rainwater retention, soil humidity, organic matter content, microbial activity, predation, and overcoming of seed dormancy. Crop rotation is an essential practice in sustainable agricultural systems because of its effects on soil fertility [36], water conservation [37], and other benefits including reduction in weed competition [38,39]. Further, mixed cropping (polycropping, intercropping) systems offer a yield buffering capacity by diverse growing demands and different periods of root, leaf, and seed development of the plant varieties. Mixed cropping systems are an efficient way to suppress weeds, particularly in competition for water and light can offer weed-suppressing capacities [40,41]. Finally, herbicides prevent some weed seeds from germinating [42] and, therefore, cut out water use by such weeds [43]; thus, good water management contributes to less weed growth, resulting in less weed density and biomass, irrespective of treatment [44].

Taking into account what has been mentioned previously, the present review has covered a great deal about the reduction in water losses caused by weeds and shows the potential of some cultural practices in interaction with reduced herbicide rates for water conservation through control of the weeds.

## 2. Material and Methods

This review is based on a structured literature search conducted in December 2024 and updated in September 2026, utilizing primary scientific databases including Google Scholar, Scopus, and Web of Science to ensure maximum coverage of peer-reviewed literature. The search was performed using specific keywords and Boolean operators to connect the main themes of the review: For weed competition and water dynamics: (“water loss” OR “moisture competition”) AND (“weed control”) AND “water conservation”. For cultural practices: (“mulching” OR “green manure” OR “cover crops” OR “crop rotation” OR “intercropping”) AND “reduced herbicide rates” AND “weed suppression”.

To select the most appropriate papers, clear inclusion and exclusion criteria were applied prior to the screening process. The inclusion criteria included: (i) peer-reviewed original research and review articles; (ii) studies focusing on the combination of cultural practices, reduced herbicide rates, and soil moisture conservation; and (iii) papers published in English between 1975 and 2026. On the other hand, the exclusion criteria involved: (i) non-peer-reviewed conference abstracts, technical reports, and gray literature; and (ii) studies testing full herbicide rates without any integration of cultural methods. The literature selection followed a step-by-step screening process. The initial database search identified a total of 540 publications. All citations were imported into Zotero reference management software (version 7.0, Corporation for Digital Scholarship), where 85 duplicate records were identified and removed. The remaining 455 unique articles were screened based on their titles and abstracts, which led to the exclusion of 215 papers that were not directly related to soil moisture or reduced-rate treatments. Finally, the full texts of the remaining 241 documents were carefully reviewed, and a final cohort of 191 publications was selected for qualitative integration in this review (Table 1).

## 3. Biophysical Mechanisms of Cultural Practices in Weed and Water Management

The exceptional physiological and morphological plasticity of dominant weeds is a decisive factor in crop–weed competition for water resources. Unlike crops, major agricultural weeds exhibit flexible transpiration regimes that allow them to maintain high biomass production even under severe moisture stress. A detailed comparative overview highlighting the biological plasticity, water consumption metrics, and root trait advantages of these major weeds compared with main agricultural crops is presented in Table 2.

To properly evaluate how cultural practices and reduced herbicide rates alter this competition and conserve water, it is essential to establish the specific agricultural and ecological meaning of various water metrics. In this context, weed water consumption represents the soil moisture directly used by weeds for their biomass production, which creates an immediate deficit for the crop. By suppressing weeds, we achieve soil water conservation, which means keeping more moisture in the soil profile by reducing evaporation from bare soil, especially when surface mulches or cover crop residues are present. This directly increases crop water availability, allowing the main crop easier access to soil moisture during critical growth stages. Consequently, higher soil moisture retention can lead to significant irrigation water savings, reducing the total amount and frequency of water that needs to be applied to the field. At the plant level, reducing weed pressure optimizes crop evapotranspiration (ET) and improves water-use efficiency (WUE). Finally, these interactions determine the yield per unit of water used, ensuring that available water contributes to higher crop production rather than supporting weed growth. These practices operate through several interconnected pathways, such as light interception and physical emergence barriers, while living covers and dead mulches block sunlight from reaching the soil surface [19,35]. This restricts the light penetration required for weed seed germination and acts as a physical barrier that delays or prevents seedling emergence [45]. Soil temperature and evaporation modification: Covering the soil regulates soil temperature fluctuations, lowering it in summer and keeping it stable [25,35]. This physical shield acts as a barrier that dramatically reduces water loss in the form of vapor and direct bare soil evaporation [19]. Improved infiltration and altered soil structure: Incorporating green manures and organic residues improves soil structure, reduces compaction, and increases soil organic matter [46]. This increases rainwater retention and accelerates infiltration, preventing surface runoff [21,35]. Competition for water and allelopathy: Suppressing weeds removes direct competition for water, shifting available soil moisture strictly to the crop [6]. Additionally, many cover crops and green manures release natural chemical compounds through roots or decomposition (allelopathy), which safely hinders weed germination without chemical inputs [27,47]. Understanding these mechanisms explains how maximizing crop water availability improves water-use efficiency [48] and increases the final yield per unit of water used [49].

## 4. Mulching and Weed Control

### 4.1. Weed Control Efficacy

Weeds compete with crops for nutrients, air, and water [50], while urbanization and global climate change will likely continue to cause limitations in water availability in some areas [51,52,53]. Mulch can address both of these problems, since application of mulch on a substrate surface can reduce, even eliminate, weeds and reduce the frequency of irrigation [20,54]. Mulches control weeds by restricting the light penetration into the soil [45,55,56], inhibiting germination and thus suppressing weed growth [57,58]. A number of studies have documented that straw mulch is an effective means of decreasing weed emergence and growth [59], conserving soil moisture [60], reducing erosion [61], and increasing soil biological activity [62]. In the study by Jodaugienė et al. [63], straw was the mulch that limited weed germination the most at the beginning of summer, by 3.5–14.1 times. In a study by Mohtisham et al. [64], straw mulch reduced the number of germinating weeds by half, compared with the non-mulched control. Mulching decreased the number of weeds in a study by Sinkevičienė et al. [46], as well. According to the authors, among the few organic mulches applied for soil mulching, straw mulch was the best for weed control. In plots with straw mulch, weed density was 2.8–6.4 times lower compared with the weed density in plots without mulch. In the study by Din et al. [49], soil mulching with wheat straw in corn cultivation contributed to a decreased mass of weeds by an average of 27.1%, compared with the plot without straw. Similarly, wheat straw and canola straw mulches decreased weed density by 84% and 79%, and weed biomass by 16 and 18%, respectively, compared with weed-infested plots [65]. In studies by Fisk et al. [66], plant mulches reduced the number of annual weeds from 41% to 78%, and weed biomass from 26% to 80% compared with non-mulched soil. Barberi & Mazzoncini [67] showed that rye reduced weed biomass from 54 to 99%, but weed growth suppression was usually higher in the years when cover crop biomass was higher. Dhima et al. [68] reported a reduction in the germination of *Echinochloa crus–galli* (L.) Beauv. (barnyardgrass) by 69% when straw mulch of barley, triticale and rye was applied in sugar beet crops. In desert pepper production, the weed population in cowpea mulch was reduced by 80% and 90%, compared with the control, for both years. Weed dry weights in cowpea mulch were 67% and 90% less than those in the control over the same period [69]. Shen & Zheng [48] investigated the efficacy of bio-based liquid mulch on weed suppression and water conservation. They concluded that weed count was reduced by at least 61% in all liquid mulched treatments compared with the control. The 1.25 and 2.0 kg/m^2^ rates were more effective for weed control than the low application rate (0.5 kg/m^2^), and there were no differences between binder loading types (AMP753 with 3.5% binder loading or AMP153 with 7% binder loading). A concise comparative summary of different organic mulch types, their application rates, and respective weed control efficacy across reported studies is presented in Table 3.

Further, the benefits of mulching and pre-emergence (PRE) applied herbicides (i.e., under the plastic) are well documented, particularly in the control of some problematic weeds [70,71,72]. For example, *Cyperus* species are among the most troublesome weeds in vegetable production in many states [73], because they readily penetrate through the plastic mulch [74]; herbicides integrated into the low-density polyethylene (LDPE) mulch–fumigant system (typically with a thickness of 25–32 μm) must control these species. Halosulfuron [75], sulfentrazone [76], and metolachlor [77] all have activity on *Cyperus* species in pepper and tomato [78]. Johnson & Mullinix [79] reported that *Cyperus esculentus* L. (yellow nutsedge) was controlled by 88% with PRE applications of halosulfuron at 36 g ai/ha underneath plastic mulch. The concentration of halosulfuron required to reduce *C. esculentus* dry weight by 90% (GR90) for PRE-applied halosulfuron, according to Adcock et al. [80], was 19.2 g ai/ha. Johnson & Mullinix [81] evaluated weed control in watermelon and cantaloupe grown on polyethylene-covered seedbeds in conjunction with various herbicide programs, including soil fumigation with metham sodium and PRE applications of either ethalfluralin alone (0.8 kg ai/ha) or ethalfluralin plus halosulfuron (0.8 kg ai/ha and 37 g ai/ha, respectively). Soil fumigation with metham–sodium followed by ethalfluralin plus halosulfuron applied PRE controlled *C. esulentus* by at least 89%. Santos et al. [82] reported that metolachlor applied pre-plant incorporated (PPI), followed by a shank-injected application of 1,3–dichlorpropene plus chloropicrin, and then followed by a post-emergence (POST) application of trifloxysulfuron, provided >90% control of *Cyperus* spp. In line with the previous study, Gilreath & Santos [83] investigated *C. esulentus* control in plastic-mulched tomatoes and reported that control was improved with a high rate of metolachlor compared with 1,3–dichloropropene (83%) or chloropicrin (17%).

### 4.2. Soil Water Conservation

Beyond the physical suppression of weeds detailed in Section 4.1, the application of surface mulch acts as a critical barrier that directly protects the soil profile [19,20]. Straw and organic mulches shield the soil surface from solar radiation, which significantly reduces evaporation losses, increases water infiltration, and reduces surface runoff [21,22,60]. By minimizing both bare soil evaporation and weed water consumption, mulching maintains a high level of crop water availability during critical dry periods, reducing the required frequency of irrigation [20,54]. This moisture retention directly improves the crop’s water-use efficiency [48]. Consequently, combining reduced herbicide rates underneath the mulch layer ensures that conserved soil water is utilized exclusively for optimizing crop evapotranspiration, thereby increasing the final yield per unit of water used [48,49].

## 5. Green Manures and Weed Control

### 5.1. Weed Control Efficacy

Green manure has been used in different agricultural systems to assist in the elimination of weeds through allelopathic effects, restricting growth space and competition for water, light, oxygen, and nutrients, and suppressing reinfestations [84,85,86]. Green manure tends to be slow-growing. However, once established, certain green manures serve the dual purpose of smothering crops, effectively outcompeting weeds and depriving them of essential resources such as sunlight, water, and nutrients [87,88]. Severino & Christoffoleti [89] evaluated the effects of the amount of green manure biomass on weed suppression and observed interactions between the amount of green manure used and the reduction in weed density and dry matter production; as the amount of green manure biomass increased, there was, in general, a reduction in weed infestation. Faba bean as green manure in concentrations of 1% and 2% of soil dry weight reduced the germination, root and shoot elongation and aerial biomass of *E. crus–galli* and *Amaranthus retroflexus* L. (redroot pigweed) by ca. 70% when the seeds were sown at the time of faba bean incorporation. In two field experiments conducted on land with a history of maize cultivation, in which faba bean biomass of 6700 and 12,100 kg/ha was incorporated into the soil, residue-mediated reductions in the density and biomass of naturally occurring dominant dicotyledonous weeds (*A. retroflexus*, *C. album*, and *Solanum nigrum* L. (black nightshade)) and monocotyledonous weeds (*Digitaria sanguinalis* (L.) Scop. (large crabgrass) and *Cyperus rotundus* L. (purple nutsedge)) were observed. At emergence, mean differences with respect to control plots ranged from −14.8% to −69.8% for density and from −46.9 to −78.5% for biomass [90]. Biennial or perennial green manures have a competitive advantage over spring-germinating weeds and can also effectively suppress some perennial weeds. For example, including perennial alfalfa has been demonstrated to reduce *Cirsium arvense* (L.) Scop. (Canadian thistle) populations, especially when the alfalfa stand is cut for forage [91]. In previous work [92,93], *Brassica kaber* (DC.) L.C. (wild mustard) growth was reduced, and interference was observed in sweet corn grown in soil amended with a *Trifolium pratense* L. (red clover) green manure and composted dairy manure compared with sweet corn grown without organic amendments but fertilized with synthetic N. Bioassays showed less weed suppression with increasing time after amendment incorporation [92], indicating that tillage timing may modify the effects of soil amendment on weeds. *Melilotus officinalis* (L.) Lam. (yellow sweet clover) residues remaining after termination of growth continued to provide excellent weed suppression. Weed densities in April before planting the succeeding wheat crop were 75 to 97% lower in *M. officinalis* than in untreated fallow treatments. *M. officinalis* controlled the perennial weeds *Taraxacum officinale* F.H. Wigg. (common dandelion) and *Sonchus arvensis* L. (perennial sowthistle), as well as *K. scoparia*, *Descurainia sophia* (L.) Webb ex Prantl (flixweed), *S. tragus* and *Bromus tectorum* L. (downy brome) [47]. Because no difference in weed suppression was observed when *M. officinalis* residues remained in the field or were removed, the authors speculated that isoflavanoids and phenolics released during growth and/or root decomposition may have explained some of the suppression. Dyck et al. [94] found that *Trifolium incarnatum* L. (crimson clover) residue reduced *C. album* biomass more than that of corn at 2 weeks after emergence (72% and 31% reductions, respectively). By final harvest, corn biomass following *T. incarnatum* had recovered to levels achieved with chemical fertilizer, while *C. album* remained 39% lower relative to conventionally fertilized treatments. In greenhouse experiments, *Brassica alba* (L.) Rabenh. (white mustard) used as a green manure crop and added to the soil at 20 g per 400 g of air-dry soil reduced the emergence of *Capsella bursa–pastoris* (L.) Medik. (shepherd’s purse), *K. scoparia*, and *Setaria viridis* (L.) P. Beauv. (green foxtail) by 97%, 54%, and 49%, respectively. Rapeseed suppressed the emergence of *C. bursa–pastoris*, *K. scoparia*, and *S. viridis* by 76%, 25%, and 25%, respectively [95]. The emergence of *E. crus-galli*, *Portulaca oleracea* L. (common purslane), *Tribulus terrestris* L. (puncturevine), or *C. album* was reduced by 11–50%, 12–59%, 26–79%, or 58–83%, respectively, in plots with aromatic plants used as incorporated green manure compared with green manure-free plots [96]. A concise comparative summary of different green manure species, their specific biomass application rates or concentrations, and their corresponding weed-suppression efficacy is synthesized and presented in Table 4.

### 5.2. Soil Water Conservation

The incorporation of green manure biomass into the soil layer not only suppresses weed density as detailed in Section 5.1, but it also alters key soil hydraulic properties. When green manure residues decompose, they significantly enhance soil organic matter content and improve soil structure [35]. This modification increases rainwater retention, improves water infiltration rates, and decreases surface runoff [35]. Furthermore, by shading the soil surface before termination, dense green manure crops act as a barrier to reduce direct water loss in the form of vapor [25]. By suppressing dominant water-wasting weeds [13,14], green manures eliminate unnecessary weed water consumption, thereby elevating overall crop water availability and soil humidity during the subsequent main cropping phase [25,35].

## 6. Cover Crops and Weed Control

### 6.1. Weed Control Efficacy

Cover crops (living mulches) are planted between the rows of a main crop such as corn, soybean, etc., and are maintained as a living ground cover during the growing season of the main crop [97]. Cover crops have the potential to form an important component in agroecosystems and can be a useful tool for weed suppression in sustainable agricultural systems [98,99,100]. They control weeds through competition [101], allelopathy [102], weed seed decay in the seed bank [103], and the proliferation of residues [92]. Water competition is another mechanism by which cover crops suppress weeds [104]. Due to their rapid early-season establishment and extensive vegetative growth, cover crops efficiently deplete available moisture in the upper soil profile, thereby restricting the water required for weed seed germination and early seedling development. Weed control when using a cover crop is dependent upon the amount of biomass on the soil surface and incorporated into the soil [105,106]. Suppression of *Amaranthus* spp., *Setaria* spp., *Ipomoea* spp., and *C. esculentus* has been successfully achieved using cover crops [107,108]. Barnes & Putnam [109] and Weston [110] reported that cover crops such as rye, barley, and wheat reduced the early season biomass of various weeds by 48 to 98%, compared with no cover crop controls. Rye and *T. incarnatum* cover crop residues reduced density of *E. crus–galli*, *Brachiaria platyphylla* (Griseb.) Nash (broadleaf signalgrass), *Brachiaria ramosa* (L.) Stapf (browntop millet), *Ipomoea hederacea* var. *integriuscula* A. Gray (entireleaf morning glory), and *Euphorbia hyssopifolia* L. (hyssop spurge), but did not affect *C. esculentus* at 7 weeks after soybean planting in the absence of herbicides [111]. Mehring et al. [112] reported that weed control in irrigated potatoes was at least 90% for all cover crop treatments (rye/canola and triticale, hairy vetch and hairy vetch/rye) and termination treatments at all three evaluation timings (14, 29, and 51 days after planting). The use of *Vicia villosa* Roth (hairy vetch) as a cover crop in temperate regions reduced weed density and weed biomass by 70–78% and 52–70%, respectively, compared with fallow treatments [113]. In addition, Teasdale and Mohler [114] showed that >75% inhibition of weed emergence is consistently achieved only when the mulch biomass and mulch thickness of *V. villosa* exceed 8000 kg/ha. According to Mohammadi [115], weed dry weight was reduced by 34 and 50.9% when *V. villosa* was interseeded in corn at 25 and 50 kg/ha, respectively. Oliver et al. [116] also reported that *V. villosa* ssp. *villosa* living mulch established in soybean reduced *Ipomoea lacunosa* L. (white morning-glory) and *Euphorbia maculata* L. (spotted spurge) biomass by about 90% and *Digitaria ischaemum* (Schreb. ex Schweig.) Schreb. ex Muhl. (smooth crabgrass) biomass by about 70% compared with weedy controls. In another study, Fujii [117] reported that complete weed control can be achieved by direct application of *V. villosa* to the rice paddy fields, while Johnson et al. [118] observed that *V. villosa* mulch completely inhibited weeds under a no-tillage system. Kunz et al. [119] found that *Sinapis alba* L. (white mustard), *Raphanus sativus* var. *niger* (Mill.) J.Kern. (black radish) and *Vicia sativa* L. (common vetch) used as cover crops suppressed *C. album*, *Matricaria chamomilla* L. (chamomile) and *Stellaria media* (L.) Vill. (common chickweed) by 60%. In the conventional system, the use of a rye cover crop resulted in a lower seedbank density compared with the crop residue treatment (–25%), whereas in the low-input system, the *Trifolium subterraneum* L. (underground clover) cover crop decreased weed seedbank density compared with the other cover crops (*T. incarnatum* and rye) and the crop residue treatment (–22% on average) [120]. Of the five cover crop systems evaluated (1-year duration), the summer fallow and the yellow mustard/buckwheat/winter rape mixture consistently reduced the weed seedbank. The magnitude of decline in these high disturbance systems reached complete depletion of the *Setaria* spp. germinable seedbank and an 85% and 80% reduction for *C. album* and *Abutilon theophrasti* Medik. (velvetleaf), respectively [121]. The combination of tillage and cover crops resulted in very high seedbank depletion rates, considerably higher than those under more conventional management. Teasdale et al. [122] reported >50% reductions in the germinable *Amaranthus hybridus* L. (smooth pigweed) seedbank in years of good weed management in a soybean cash crop. In another study that included eight locations across the US Corn Belt, *C. album*, *Setaria faberi* Herrm. (giant foxtail) and *A. theophrasti* seedbanks declined 40–47%, 78% and 42%, respectively over a 1-year period [123]. A comprehensive comparative summary of diverse cover crop species, their corresponding biomass rates or seedbank disturbance intensities, and their respective weed-suppression efficacy is synthesized and presented in Table 5.

The allelopathic properties of cover crops can also be used to control weeds. For example, the allelopathic properties of *Secale cereale* L. (rye), *Lolium* spp., and *T. subterraneum* can be used to control weeds in sweet corn and snap beans [124]. Special weed control properties of winter turnip rape and other Brassica plants have been highlighted by Al–Khatib et al. [95], Petersen et al. [125], and O’Reilly et al. [126]. They are associated with the fact that these plants secrete isothiocyanates, which are toxic to some weed species. It has been demonstrated that winter turnip rape secretes substances that hinder seed sprouting of *Sonchus asper* (L.) Hill (prickly sowthistle), *Matricaria inodora* L. (scentless chamomile), *A. hybridus*, *E. crus–galli* and *Alopecurus myosuroides* Huds. (black-grass) [125]. In most cases, the utilization of allelopathic cover crops involves the use of their residues as surface mulch or the application of their extracts as post-emergence sprays in combination with reduced herbicide dosages [127,128]. The use of a rye cover crop with half the recommended rate of atrazine plus metolachlor in sweet corn resulted in excellent control of *A. retroflexus* and *C. esculentus* in field experiments in Arkansas [129]. Wallace & Bellinder [130] reported that a no-till rye cover crop in conjunction with reduced rates of linuron, metolachlor, and metribuzin provided >90% *A. retroflexus* control and >93% control of *C. album* in sweet corn, snap beans, potato, and tomato. Wild radish or rye cover crops in conjunction with a half or full rate of atrazine and S–metolachlor controlled >95% *Richardia scabra* L. (rough Mexican clover), *I. hederacea,* and *D. sanguinalis* in sweet corn [131]. A multiplicative model that included a quadratic response to *V. villosa* residue and a log–logistic response to metolachlor effectively showed that emergence of *A. hybridus*, *C. album*, *S. faberi*, and *A. theophrasti* and early growth of *A. hybridus* and *C. album* were reduced synergistically. For example, *A. hybridus* emergence was reduced by 13% by 500 g/m of *V. villosa* residue alone and by 16% by 10 g/ha of metolachlor alone, but together, they reduced *A. hybridus* emergence by 86% [132].

### 6.2. Soil Water Conservation

While living cover crops suppress weeds through intense resource competition as described in Section 6.1, their residues serve a vital protective function for soil water conservation. Once terminated, the dense proliferation of cover crop residues left on top of the soil forms a stable surface mulch [127]. This physical barrier protects the soil surface against weather aggressions and water erosion [31], while lowering soil temperatures and reducing moisture losses via evaporation [32,35]. By dramatically depleting weed seedbanks and minimizing early weed competition [121,123], integrated cover crop systems paired with reduced herbicide rates ensure that saved moisture is fully redirected toward crop water availability, maximizing final water productivity [32,132].

## 7. Crop Rotation and Weed Control

### 7.1. Weed Control Efficacy

Crop rotation may be an effective practice for controlling serious weeds because it introduces conditions that affect weed growth and reproduction, which may greatly reduce weed density [133,134,135]. Dissimilar crop species with disparate management practices impose a wide range of stresses and mortality factors, creating an unpredictable environment to which the weed community is continually adjusting [136]. In addition, weed diversity has been shown to increase under crop rotation compared with monoculture [137,138]. It has also been suggested that weed densities are lower in crop rotation systems than in monocultures [139]. For example, the weed population in continuous winter wheat plots comprised 90% grass and sedge weeds, while in sugar beet–wheat rotation, it was only 43% of total weed density. Broadleaf weeds accounted for 55.2% of the total weed density in continuous winter wheat, but only 9.4% in sugar beet–winter wheat [140]. Furthermore, crop rotations have been shown to maintain or decrease the number of weed seeds in the soil [141,142]. Forcella & Lindstrom [143] reported that after seven to eight years of weed management, the number of weed seeds was about six times greater in continuous corn production than in a rotated corn–soybean system. Soybean–corn crop rotation reduced in–field and seedbank *Conyza canadensis* (L.) Cronquist. (Canadian horseweed) densities compared with continuous soybean in the third and fourth growing years [144]. Similarly, Covarelli & Tei [145] found three times as many seeds in the seedbank of continuous corn plots compared with wheat–corn rotational plots. The integration of *T. pratense* in the sweet corn–peas–wheat rotation led to a 96% reduction in seedbank density of winter annuals [146]. Recommendations for control of *Aegilops cylindrica* Host (jointed goatgrass) in winter wheat in the Pacific Northwest include growing a spring wheat crop in the rotation to reduce the soil seedbank [147]. In the past, crop rotations involving spring or summer crops presented the best solution for controlling *B. tectorum* in winter wheat in Nebraska [148]. Additional research in Nebraska demonstrated that a 3–year winter wheat–fallow rotation that included a summer crop reduced *B. tectorum*, *A. cylindrica*, and feral rye [149]. By succeeding wheat with a clean winter fallow before sowing a summer crop, almost complete control of *Avena fatua* L. (wild oat) seedlings can be achieved, thereby eliminating seed production. Thus, as found by Philpotts [150], *A. fatua* and *Avena sterilis* ssp. *ludoviciana* (Durieu) Nyman (winter wild-oat) populations could be dramatically reduced by winter-fallowing in association with the rotation of wheat and sorghum crops. One winter fallow reduced the population by 96.6% and two winter fallows reduced it by 99.8%. Similarly, Martin and Felton [151] showed that the rotation of wheat with sorghum, which allowed two winters for the control of *A. fatua* either by cultivation or with herbicide, resulted in an exponential decline to low numbers of *A. fatua* seed in the soil. The *Bromus secalinus* L. (rye brome) management programs in the Southern Great Plains, according to Stone et al. [152], initiated following harvest of winter wheat, included conventionally tilled, double-crop grain sorghum followed by soybean. The results showed that *B. secalinus* panicles, as well as dockage due to *B. secalinus* in the succeeding wheat, were reduced by up to 87%, while *B. secalinus* panicles and dockage were reduced by up to 78% and 87%, respectively, by rotation out of winter wheat for one growing season. Similarly, Anderson et al. [153] investigated alternative crop rotations, with the goal of replacing winter wheat–fallow in the semiarid Great Plains. According to the results, the rotations with the lowest number of weed seedlings were winter wheat—fallow and spring wheat—winter wheat—corn—sunflower; in comparison, weed density was six–fold higher in winter wheat—proso millet. The density of *B. tectorum* and *K. scoparia* was highest in winter wheat—proso millet compared with the other rotations, whereas *Eragrostis cilianensis* (All.) Vignolo ex Janch. (stinkgrass) and *S. viridis* were prominent in proso millet of the winter wheat—proso millet and winter wheat—corn—proso millet rotations. Replacing wheat with crops such as *Trifolium alexandrinum* L. (Egyptian clover), potato and oilseed rape for a 2–3-year period in a rice–wheat cropping system significantly reduced the population of *Phalaris minor* Retz. (littleseed canarygrass) [154]. In another study, the inclusion of alfalfa in the crop rotation sequence significantly decreased the interference of weeds in the subsequent crops [155]. Ominski et al. [156] conducted a survey in 117 fields in Manitoba, Canada, and found that rotation with alfalfa can effectively reduce the interference of *A. fatua*, *C. arvense*, *B. kaber* and *Galium aparine* L. (cleavers) in the subsequent cereal crops. Crop rotation had a significant effect on the growth of *E. crus–galli*, *Sagittaria sagittifolia* L. (arrowhead), and *Alisma plantago–aquatica* L. (common water-plantain) weeds. According to Filizadeh et al. [157], continuous rice planting produced the highest density of these weeds, compared with a rice–soybean–rice rotation. There were 62.5% and 80% reductions in weed density and weed biomass, respectively, in the rice–soybean–rice rotation compared with continuous rice planting.

Another benefit of crop rotation may be associated with a lower chance of selecting troublesome weeds, because the crop rotation sequence also determines herbicide use, and crop rotation and herbicide use can interact to affect weed species [158]. Therefore, the practice of rotating crops and herbicides has proved to be successful in influencing weed populations and improving crop production [159], and, given the increased attention paid to agroecosystem biodiversity, adopting weed management strategies that promote weed species diversity could be encouraged [160]. In an experiment with corn, soybean, and wheat, Doucet et al. [161] reported that combined use of crop rotations and herbicide management resulted in more effective weed control than either rotation or herbicide treatment alone. The results of the study by Heggenstaller & Liebman [162] indicated that rotations including triticale and lucerne with reduced rates of herbicides can facilitate the suppression of *A. theoprasti*. Rotations that include lucerne can contribute to restraining *S. faberi* population growth, given adequate levels of seedling mortality in this crop. Mean weed biomass in corn and soybean was <25 kg/ha in all rotation × herbicide combinations except the low-herbicide 3-year rotation (corn–soybean–oat/red clover sequence), which contained ∼110 kg/ha of weed biomass [163].

### 7.2. Soil Water Conservation

Beyond creating an unpredictable selection pressure for the weed community detailed in Section 7.1, alternating crops with diverse root structures optimizes soil moisture conservation. Crop rotation sequences alter the depth and intensity of root moisture extraction from the soil profile, which prevents continuous localized water depletion [37]. Incorporating specific rotational choices, such as deep-rooted perennial alfalfa or clean winter fallows, significantly improves water infiltration and breaks weed reproductive cycles [155]. By maintaining low weed seedling densities, crop rotations reduce the total volume of water wasted by weed populations [13,14,139]. This synergistic interaction between diverse crop cycles and low-input herbicide programs ultimately secures long-term crop water availability and yield stability [161,162]. Crop rotation serves a foundational role in soil water conservation through several distinct biophysical mechanisms as well. First, alternating crop species with different rooting depths optimizes moisture extraction across the entire soil profile; deep-rooted crops (such as alfalfa) draw water from lower layers, preventing localized depletion and opening soil pores that improve subsoil capillary action [46,155]. Second, the alteration of crop phenology and growing periods shifts the timing of canopy coverage and peak water demand, minimizing direct soil water evaporation during high-radiation months [37]. Third, diverse crop cycles significantly enhance soil structure by reducing soil compaction and creating continuous macropores, which accelerates rainwater infiltration and reduces surface runoff [46]. Fourth, the systematic accumulation of diversified plant residues elevates soil organic matter, which bonds soil aggregates together and exponentially increases the soil’s water-holding capacity [46,49]. By maintaining low weed seedling densities, crop rotations reduce the total volume of water lost through weed transpiration [13]. This combined improvement in soil physics and reduction in weed pressure ultimately enhances crop water-use efficiency and secures long-term yield stability [37,161].

## 8. Intercropping and Weed Control

### 8.1. Weed Control Efficacy

Intercropping has certain benefits in terms of production [164], soil health [165], water conservation [166] and system productivity [167]. In addition, intercrops offer significant assistance in weed control due to enhanced competition, physical dominance, space occupation, and allelopathic influence [41,168,169,170]. The addition of another crop species between rows can lead to smothering of and greater competition with weed species, minimizing their impact on the main crop being cultivated [171]. Intercrop treatments such as wheat–canola and wheat–canola–pea tended to provide greater weed suppression compared with each component crop grown alone, indicating some kind of synergism among crops within intercrops with regard to weed suppression [40]. Naeem et al. [172] found that mixed cropping of wheat + canola suppressed the dry weight of *P. minor*, *C. album*, *Rumex dentatus* L. (toothed dock) and *Coronopus didymus* (L.) Sm. (lesser swinecress) by 94%, 77.2%, 77.4% and 92%, respectively, compared with sole wheat cropping. The other intercropping treatments, such as one row of wheat + one row of canola, two rows of wheat + two rows of canola, and four rows of wheat + four rows of canola generally suppressed total dry weight of weeds by 81%, 74% and 76%, respectively. A significant reduction in weed density and biomass in wheat/chickpea intercrops compared with both sole wheat and sole chickpea crops was found by Banik et al. [173]. Strip intercropping of maize, common bean, and spring wheat markedly reduced the number of *Galinsoga parviflora* Cav. (gallant soldier), *E. crus–galli*, *C. album* and *A. retroflexus* per unit area (by 50%), as well as the dry weight of their aerial parts [174]. Mixed cropping of peas with false flax in additive arrangements had a strong suppressive effect on weed coverage, i.e., 63% in the first and 52% in the second year, compared with sole pea cropping [175]. Taking linseed as the target crop for weed reduction, intolerable weed cover of 34% and 54% in pure stands was significantly reduced in a mixture with wheat to an acceptable level of 10% by increasing the crop cover [176]. Field trials in Pakistan indicated that intercropping single and double rows of sorghum, soybean and sesame in a cotton crop reduced *C. rotundus* density by 70–96% and its dry matter production by 71–97% [177]. Cotton + sunflower intercropping reduced total weed growth by 85% [117] and produced higher cotton equivalent yields and net income [178]. Intercropping sorghum with maize significantly reduced density and biomass of *C. rotundus*, *Convolvulus arvensis* L. (field bindweed), and *Trianthema portulacastrum* L. (desert horsepurslane) [179]. Intercropping of sorghum and sunflower with cotton suppressed weeds significantly by 62.4% and 59.6%, respectively, and significantly enhanced growth and yield traits of cotton, particularly seed cotton yield, in comparison with no weeding [180]. Bilalis et al. [181] reported that intercropping maize with legumes considerably reduced weed density in the intercrop compared with a maize sole stand due to decreased light availability for weeds in the maize–legume intercrops, which led to a reduction in weed density and weed dry matter compared with sole crops. Recently, it was demonstrated that soybean–maize intercropping significantly diminished the biomass of *A. retroflexus* and *Datura stramonium* L. (jimsonweed) [182]. Intercropping systems with leguminous species are particularly beneficial for farmers struggling with *Striga asiatica* (L.) Kuntze (Asiatic witchweed) or *Striga hermonthica* (Delile) Benth. (purple witchweed) infestations: one 7-year study in Kenya determined that several edible legume species, including crotalaria and groundnut, reduced *S. hermonthica* emergence by up to 35% [183]. A more recent study by Midega et al. [183] indicated that drought-tolerant *Desmodium uncinatum* (Jacq.) DC. (silverleaf desmodium) and *Desmodium intortum* (Mill.) Urb. (greenleaf desmodium) significantly reduced *Striga* spp. incidence in sorghum–Desmodium intercropping systems. Intercropping cassava with compatible crops is one of the effective approaches to weed management in this crop [184,185]. Osundare [186] recorded weed densities of 94, 96, 97, and 96 plants/m^2^ for cassava–cowpea, cassava–peanut, cassava–pigeon pea, and cassava–soybean combinations, respectively, compared with 132 plants m/^2^ for sole cassava. Zuofa et al. [187] reported a 13% reduction in weeds in cassava–maize intercropping, and a higher reduction of 16–40% when slow-growing smother crops such as peanut, cowpea, or melon were intercropped with cassava. Moreover, compared with a cassava sole crop, an okra–cassava intercrop decreased weed competition by around 40–45% [188], while a cassava–pumpkin intercrop decreased weed growth by 70–100% [189]. In principle, weed suppression has often been found to be greater in intercrops compared with sole crops. For example, Liebman & Dyck [190], in an extensive literature review, reported that weed biomass in intercrops was lower than that in component crops in 50% of the studies, intermediate to component crops in 42% of the studies, and greater than that in all component crops in 8% of the studies. Therefore, the strategic selection of intercropping components remains critical to maximize spatial canopy coverage and exploit crop-specific traits, as demonstrated by the high efficacy of cereal–legume combinations (e.g., maize–bean–wheat or wheat–chickpea) in successfully outcompeting aggressive annual weeds [173,174].

### 8.2. Soil Water Conservation

The physical dominance and rapid space occupation of intercropping systems establish a highly efficient canopy for water conservation. Cultivating complementary crop species between the rows leads to faster ground cover, which minimizes direct light interception at the soil surface and cuts down soil water evaporation [40,41]. This spatial niche differentiation allows the mixed root systems to optimize water uptake from different soil depths, avoiding direct moisture stress [40,166]. By working together as a synergistic filter against aggressive, water-consuming weeds [13,14,40], intercrops protect existing soil moisture reservoirs. The resulting reduction in overall weed biomass ensures that crop evapotranspiration remains efficient, leading to higher system productivity per unit of water used [166,167].

## 9. Trade-Offs and Practical Limitations of Integrated Cultural Practices

While integrating cultural practices with reduced herbicide rates offers clear benefits for weed control and soil water conservation, several practical trade-offs and limitations must be considered. First, financial and labor costs represent a major constraint. Practices like spreading mulches, establishing cover crops, or shifting to complex crop rotations require significant initial investments, specialized farm machinery, and higher management labor compared with standard herbicidal-only programs [19,136]. Second, living cover crops and green manures can sometimes create a direct conflict for resources. In strictly arid or dryland conditions during years of extreme water shortage, a living cover crop can consume the limited soil moisture profile intended for the main cash crop before termination, leading to early-season crop water stress [32,104]. Additionally, a high accumulation of crop residues can lower soil temperatures in early spring, which may delay seed germination and early development of warm-season crops like corn or cotton [35,114]. Furthermore, heavy residue layers can significantly reduce the efficacy of PRE herbicides. These herbicides often bind strongly to the organic matter of the residues, preventing them from reaching the soil surface where they are required to form an effective herbicide film and manifest their weed control activity [40,132]. Lastly, variable weather conditions, especially the scarcity of precipitation after preemergence herbicide application, can alter both residue decomposition and herbicide activation, making weed management outcomes unpredictable [40]. Acknowledging these practical constraints is essential for farmers to correctly implement these integrated strategies based on their local climate and economic capacity. Finally, a critical conceptual constraint involves the discussion of herbicide resistance management. Reduced herbicide rates must not be recommended universally. Applying sub-lethal herbicidal rates can accelerate the evolution of metabolic non-target-site resistance (NTSR) in cross-pollinated weed populations, as it allows individuals with low-level tolerance to survive and reproduce. Therefore, reduced herbicide rates should never be used as an unintegrated, single strategy. Instead, they must be strictly integrated with diversified cultural practices—such as crop rotation, cover crop residues, and alternating herbicide modes of action—which act as non-chemical resistance-management tools. Cultural methods lower the initial weed density, ensuring that any subsequent application of a reduced herbicide rate successfully prevents seedbank replenishment without driving rapid resistance selection. Furthermore, the biochemical nature of the utilized biomass plays a foundational role in modulating the duration of weed suppression. Residues characterized by a high lignin and cellulose content, such as wheat straw or chemically terminated mature rye, undergo significantly slower microbial degradation in the field [47,67]. This persistent nature sustains the physical emergence barrier and prolongs the inhibition of weed germination over an extended timeframe. Conversely, succulent green manures and leguminous cover crops (such as faba bean or hairy vetch) feature a low carbon-to-nitrogen (C:N) ratio and minimal lignin protection [90,114]. Consequently, they decompose at an accelerated rate, delivering an intense but short-lived suppressive or allelopathic effect. Managing this degradation window is a critical trade-off, as crop-wise weed control success depends on matching the residue persistence with the critical weed-free period of the main crop, as summarized in Table 6.

## 10. Future Horizons: Precision Weed Management and Water Optimization

As agriculture shifts towards sustainability, the future of optimizing the interaction between cultural practices and reduced herbicide rates lies in precision weed-management technologies. Modern digital tools, including AI-driven site-specific weed recognition, automated mechanical weeding, and variable-rate herbicide application systems, offer unprecedented opportunities to minimize chemical loads while maximizing resource efficiency. Integrating these advanced technologies with soil-water sensor arrays allows farmers to apply micro-doses of herbicides only when weed patches directly threaten soil moisture reserves. Adopting these next-generation precision frameworks will be essential to scale up soil water conservation strategies and safeguard crop yields against volatile climate shifts under real field conditions [191]. A comprehensive summary of the integrated cultural practices, reduced herbicide rates, and their corresponding effects on weed suppression, soil hydrology, and crop performance is synthesized and presented in Table 7. Furthermore, a direct quantitative comparison of these five agricultural practices, outlining their long-term effectiveness, climatic suitability, target weed groups, required biomass rates, feasible herbicide reductions, and final yield stability, is comprehensively synthesized and presented in Table 8.

## 11. Conclusions

Efficient weed control is essential for sustainable agriculture, offering numerous benefits, including water conservation. Based on the reviewed literature, mulching and cover crop residues exhibit the strongest and most consistent empirical evidence for achieving simultaneous weed suppression and direct soil water conservation. Conversely, evidence remains insufficient regarding the long-term economic scalability of green manures in strictly arid dryland farming and the exact multi-species weed thresholds under complex crop rotations. In terms of chemical optimization, combining these cultural practices with low-input management makes a 30% to 50% reduction in post-emergence and pre-emergence herbicide rates highly realistic and feasible under real field conditions, without compromising final crop yields. However, reduced rates must not be recommended universally as an unintegrated, single chemical strategy to avoid the risk of metabolic resistance selection. Ultimately, a holistic approach that integrates these multifunctional cultural practices with targeted precision technologies is essential to optimize water productivity, safeguard agroecosystem stability, and ensure long-term agricultural sustainability.

## Figures and Tables

**Table 1:** Number of publications included in the qualitative review according to the main weed control approach and its relevance to water conservation.

Type of Study	Number of References
Loss of water caused by weeds	16
Weed control by mulching and herbicides	41
Weeds suppressed by green manure	20
Weeds suppressed by cover crops and herbicides	46
Control of weeds by crop rotation and herbicides	35
Control of weeds by intercropping	32
Control of weeds by precision technologies	1

**Table 2:** Comparative evaluation of physiological plasticity, water consumption, and root traits between major weeds and agricultural crops.

Plant Species	Category	Water Requirement/Consumption Ratio	Key Physiological Plasticity and Root Morphology	Reference
*Chenopodium album*	Weed	658 lbs of water per 1 lb of dry matter	High drought tolerance, outcompetes crop for moisture	[12]
*Amaranthus retroflexus*	Weed	912 lbs of water per 1 lb of dry matter	High biological plasticity, rapid growth in dry years	[12]
*Sinapis arvensis*	Weed	Consumes as much moisture as 4 wheat plants	Rapid early-season vegetative establishment	[15]
*Salsola tragus*	Weed	Consumes as much moisture as 3 sorghum plants	Extremely rapid root development; reaches deep moisture first	[16]
Corn (Maize)	Crop	349 lbs of water per 1 lb of dry matter	Sensitive to early-season crop–weed moisture stress	[12]
Wheat	Crop	557 lbs of water per 1 lb of dry matter	Limited root plasticity under heavy weed infestations	[12]

**Table 3:** Comparative evaluation of organic mulch types, biomass application rates (kg/ha, kg/m^2^, or %), and weed suppression efficacy.

Mulch Type	Biomass Application Rate/Intensity	Target Weed Population/Metrics	Weed Control Efficacy	Reference
Straw Mulch (General)	Standard surface cover	General annual weed germination	Reduced germination by 3.5–14.1 times	[63]
Wheat/Canola Straw	Surface layer	Weed density and mass reduction	79% to 84% lower density; 27.1% biomass mass reduction	[49,65]
Straw Mulch (Best Organic)	Surface placement	Total weed density per plot	2.8 to 6.4 times lower density compared with control	[46]
Rye/Barley/Triticale Straw	Crop residue matrix	*Echinochloa crus-galli* in sugar beet	69% reduction in early germination	[68]
Cowpea living mulch	Cover biomass return	Weed population density and dry weight	80% to 90% weed population reduction; up to 90% dry weight reduction	[69]
Bio-based liquid mulch	High loading rate (1.25 and 2.0 kg/m^2^)	Total weed count	At least 61% reduction in all treated plots	[48]

**Table 4:** Comparative evaluation of green manure types, application rates (kg/ha or % of soil dry weight) and respective weed suppression efficacy.

Green Manure Species	Application Rate/Biomass Concentration	Target Weed Population/Metrics	Weed Control Efficacy	Reference
Faba bean	1% and 2% of soil dry weight	*Echinochloa crus-galli*, *Amaranthus retroflexus*	~70% reduction in germination, root/shoot elongation, and biomass	[90]
Faba bean residues	6700 and 12,100 kg/ha (soil incorporated)	Dominant broadleaved and grass weeds	Density reduced by 14.8% to 69.8%; Biomass reduced by 46.9% to 78.5%	[90]
Perennial alfalfa	Rotational forage stand cuts	*Cirsium arvense*	Significant population reduction and long-term suppression	[91]
*Melilotus officinalis*	Growth residues remaining post-termination	*Taraxacum officinale*, *Sonchus arvensis*, *Kochia scoparia*	75% to 97% lower weed densities prior to subsequent wheat sowing	[47]
*Trifolium incarnatum*	Field incorporation residue layer	*Chenopodium album*	72% biomass reduction at 2 weeks; 39% reduction at final harvest	[94]
*Brassica alba*	20 g per 400 g of air-dry soil (5% *w*/*w*)	*Capsella bursa-pastoris*, *Kochia scoparia*, *Setaria viridis*	Emergence reduced by 97%, 54%, and 49%, respectively	[95]
Rapeseed	Standard soil amendment layer	*Capsella bursa-pastoris*, *Kochia scoparia*, *Setaria viridis*	Emergence suppressed by 76%, 25%, and 25%, respectively	[95]
Aromatic plants	Incorporated green manure volume	*Echinochloa crus-galli*, *Portulaca oleracea*, *Tribulus terrestris*, *Chenopodium album*	Emergence reduced across various high ranges (up to 83% for *Chenopodium album*)	[96]

**Table 5:** Comparative evaluation of cover crop types, biomass application rates (kg/ha or kg seed/ha), and respective weed suppression efficacy.

Cover Crop Species	Biomass Rate/Seeding Intensity	Target Weed Population/Metrics	Weed Control Efficacy	Reference
Rye, Barley, Wheat	Living ground cover residues	Early season annual weeds	48% to 98% reduction in early season biomass	[109,110]
Rye/Trifolium incarnatum	Surface crop residues	*Echinochloa crus–galli*, *Brachiaria platyphylla*, *Brachiaria ramosa*, *Ipomoea hederacea*, var. *integriuscula*, *Euphorbia hyssopifolia*	Marked density reduction, but no effect on *Cyperus esculentus*	[111]
Rye/Canola, Triticale, Hairy Vetch	Irrigated system termination	General field weeds in potatoes	At least 90% control maintained up to 51 days	[112]
*Vicia villosa*	Biomass exceeding 8000 kg/ha	Emerged annual weeds	Consistent >75% inhibition of weed emergence	[114]
*Vicia villosa* (interseeded)	25 and 50 kg/ha seeding rates	General weeds in corn	Weed dry weight reduced by 34% and 50.9%, respectively	[115]
*Vicia villosa* ssp. *villosa*	Living mulch in soybean	*Ipomoea lacunose*, *Euphorbia maculata*, *Digitaria ischaemum*	Biomass reduced by 70% to 90% compared with controls	[116]
*Sinapis alba*, *Raphanus sativus*, *Vicia sativa*	Biomass canopy coverage	*Chenopodium album*, *Matricaria chamomilla*, *Stellaria media*	60% suppression of target weed species	[119]
Yellow mustard/Buckwheat/Winter rape	High disturbance mixture (1-year)	Germinable seedbank	Complete depletion of *Setaria* spp.; 80–85% reduction for *Chenopodium album* and *Abutilon theophrasti*	[121]

**Table 6:** Breakdown of biomass types, reported timeframes for weed suppression, and the influence of lignin-mediated degradation rates.

Biomass Material	Main Cash Crop	Reported Timeframe for Active Weed Suppression	Biomass Nature and Lignin Influence on Degradation	Reference
Wheat/Canola Straw	Corn/Vegetables	Full early season (critical weed-free period)	High lignin and cellulose content; slow microbial degradation sustains a lasting physical barrier.	[49,65]
Cereal Straw (Rye/Barley)	Sugar Beet/Vegetables	Early summer phase (up to 3.5–14.1 times reduction at start)	Moderate to high lignin; resists rapid breakdown under variable moisture conditions.	[63,68]
Faba Bean (Green Manure)	Maize land history	Initial emergence phase (reductions from emergence onwards)	Low lignin, high nitrogen content; decomposes rapidly, offering early allelopathic flash but short physical cover.	[90]
Hairy Vetch/Rye Mix	Irrigated Potatoes	Up to 51 days post-planting (evaluated at 14, 29, and 51 days)	Mixed biomass; the high-lignin rye slows down the rapid degradation of the succulent vetch	[112]
*Vicia villosa* residues	Soybean/Corn	Up to 7 weeks after cash crop planting	Moderate degradation speed; thickness above 8000 kg/ha is required to offset natural breakdown	[111,114]
*Melilotus officinalis*	Subsequent Wheat	Extended winter/spring window (evaluated in April prior to sowing)	High phenolic and structured tissue content; remains effective for months after chemical termination	[47]

**Table 7:** Overview of integrated cultural practices and reduced-rate herbicide interactions on weed control, soil water conservation, and crop yield.

Cultural Practice	Crop	Weed Species	Practice Rate/Intensity	Herbicides Used	Herbicides Reduction	Weed Control Response	Soil Water Response	Yield Response
Plastic/Polyethylene Mulch	Pepper/Tomato	*Cyperus esculentus*	Low-density polyethylene (LDPE)	Halosulfuron/Metolachlor	Up to 50% reduction underneath plastic	>88% control of *Cyperus* spp.	Significantly reduced evaporation	Improved crop establishment
Cover Crops (Rye residues)	Sweet Corn	*Amaranthus retroflexus*, *Chenopodium album*	Surface mulch biomass	Atrazine + Metolachlor	50% of recommended field rate (0.5×)	Excellent suppression (>90% control)	Maintained soil moisture profiles	Stable commercial yields
Cover Crops (*Vicia villosa*)	Corn	*Amaranthus hybridus*, *Setaria faberi*	500 g/m^2^ biomass density	Metolachlor	Synergy with ultra-low micro-rates	86% emergence reduction (Synergistic effect)	Retained early season moisture	Increased vegetative biomass
Crop Rotation (Lucerne/Triticale)	Corn/Soybean	*Abutilon theophrasti*, *Setaria faberi*	3-year rotational sequence	Rotational chemistry	Variable low-input chemical programs	Marked suppression of seedbank and plants	Enhanced deep soil water profiles	Long-term yield stability
Intercropping (Wheat + Canola)	Wheat	*Phalaris minor*, *Chenopodium album*	Mixed additive arrangements	Targeted postemergence entries	Reduced reliance on full herbicide cover	77% to 94% dry weight suppression	Optimized spatial water-niche use	Higher land equivalent ratio (LER)

**Table 8:** Overview of cultural practices combined with reduced herbicide rates across climatic zones and weed groups, biomass application intensities (kg/ha or relevant thresholds), and impact on crop yield.

Cultural Practice	General Effectiveness	Optimal Climatic Conditions	Target Weed Groups	Typical Biomass/Application Rates	Feasible Herbicide Reduction	Impact on Crop Yield
Mulching	Very High (consistent suppression)	Arid, semi-arid, and temperate zones	Annual grasses, sedges, broadleaves	Varies by material (organic layer or synthetic film)	50% or more (underneath mulch barrier)	Maintained or increased (due to moisture retention)
Green manures	High (variable based on biomass quantity)	Temperate and sub-humid zones	Early spring broadleaves, select annual grasses	1% to 2% soil dry weight (approx. 5000–12,000 kg/ha)	30% to 50% (via residual allelopathy)	Maintained (long-term improvement of soil fertility)
Cover crops	High to Very High (biomass dependent)	Temperate, humid, and semi-arid irrigation areas	Diverse annual broadleaves, germinating weed seeds	Exceeding 8000 kg/ha for consistent >75% suppression	50% reduction (demonstrated micro-dose synergy)	Maintained (after cover termination)
Crop rotation	Moderate to High (cumulative over long term)	Semi-arid, Mediterranean, and temperate zones	Serious perennial weeds, aggressive single-crop monoculture specialists	2- to 3-year multi-crop rotational sequence	Rotational reduction (low-input chemical entry alternate)	Maintained and Stabilized (yield buffering effect)
Intercropping	High (due to rapid spatial canopy coverage)	Sub-humid, temperate, and tropical farming areas	Broadleaves, problematic parasitic weeds (*Striga* spp.)	Additive strip row arrangements/higher crop cover density	30% to 50% (via enhanced initial spatial competition)	Increased (higher system productivity and net income)

## Data Availability

No new data were created in this study. Data sharing is not applicable to this article.
